# Experimental Researches on the Properties and Functions of the Nervous System, in Vertebrated Animals

**Published:** 1831

**Authors:** P. Flourens


Art. II.—*Experimental Researches on the Properties and Functions of the Nervous System, in Vertebrated Animals.* By P. Flourens.—Translated from the French. By the Junior Editor.

Since the interesting work of Mr. Charles Bell, in which, by a simplicity of experiment, and an ingenuity of reasoning, seldom surpassed, he has established the different origin of the nerves of sensation, locomotion, and respiration, we have seen nothing on the subject more interesting, from its novel and curious research, than the work now before us. The author may be said to have pushed the investigations to which the discoveries of Mr. Bell so evidently led the way, into a much more extended field.

It is known even to the vulgar, that the nervous system in the animal economy, is subservient to sensation and motion, as well as to the intellectual faculties. But whether the exercise of these functions is to be referred to any peculiar part of the nervous system, or whether they reside in common, in the same part of the brain, had never been satisfactorily ascertained, until the experiments of our author. So long as physiologists confined themselves to abstract discussion, and vague analogies, the solution of the question was unsatisfactory to the great majority of readers. Experiments like those of the author, appreciable by all, and subject to no inaccuracy, could alone determine the fact.

So interesting is the nature of these experiments, that, horrible as they are from their cruelty, to all but confirmed vivisectors, we shall proceed to translate such parts of the author's work, as will give our readers an idea of the additions he has made to our stock of physiological knowledge.

In the course of his investigations, the author appears to have established very satisfactorily the following facts:—The nerves, the spinal marrow, and the tubercles, produce muscular contraction, the cerebral lobes are the seat of the will and sensation, and the cerebellum possesses a property which has not hitherto been recognized in physiology, viz., that of combining the motions of the muscles, in those movements in which joint action of different sets of muscles is necessary.

We have then three distinct parts of the nervous system, presiding over as many distinct functions. 1. The cerebrum, the organ of perception, volition, and the intellectual faculties. 2. The cerebellum combining and regulating those muscular movements, whose concerted action is necessary in locomotion, prehension, &c. 3. The spinal marrow, of which the *medulla*

*oblongata* is a part, and the nerves, the immediate agent of muscular contraction. An amimal deprived of its cerebral lobes loses its sensibility, and its intellectual faculties; but preserves its locomotive and prehensive faculties, unimpaired; deprived of the cerebellum, it preserves its sensations, but the muscles refuse to act in concert.

Not only does sensation and motion depend upon different parts of the brain, but sensation and the senses have a distinct location also. The ablation of the cerebral lobes destroys the sight immediately, but the iris still moves, showing that the retina retains its sensibility. The ablation of the tubercles at once destroys the mobility of the iris, showing that the optic nerve has lost its function. By the one operation, we destroy the sensation of seeing, by the other, the sense of sight.*

It was more difficult to ascertain whether the animal, by the destruction of the cerebral lobes, lost all the other senses. "The only suitable means," says the author, "of ascertaining this fact, but which in his opinion is infallible, is to preserve

---

* The author, we think, has here fallen into an error. The organ which presides over sensation must, of consequence, preside over the senses also, which are but a modification of the former. The tubercles are the commencement of the spinal marrow, and consequently like it, the origin of muscular motion, as the author has clearly shown. When the brain is destroyed, the iris plays when an impression is made upon the retina, because the source of its motion is untouched; but when we wound the tubercles, we cut off the communication of the iris with the brain, and thus deprive it of its mobility, by destroying the source of muscular power. Even this event would not immediately take place, if the iris contracted under the direct impression of the rays of light, for the nerves retain the power of exciting muscular contraction, after their communication with the brain is cut off. It is probable that the sympathetic actions, like those of the iris, require the co-operation of some part of the brain, and hence, in order to their taking place, it is necessary that the origin of the nerve connecting them with the brain, should be entire, both that its relation with the rest of the nervous system may be unimpaired, and that it may carry back the cerebral influence to the sympathising muscle. It is therefore clearly wrong to speak of the remaining *sensibility* of the retina, unless by sensibility he understands the power of producing muscular action, which a nerve possesses independent of the brain. This signification, however, is an abuse of terms, which, however it may be sustained by the poverty of the French and English languages, has no precedent in either.

the animal as long as possible after the operation. The animal, once cured of the mechanical injuries which would accompany the ablation of the cerebral lobes, would recover by degrees those faculties which do not essentially depend upon these lobes."

The experiments which he has undertaken with this view, and which form the subject of the second memoir in this work, plainly show, that so long as the animals survive the ablation of these lobes, (and he has seen them survive a whole year,) they remain constantly asleep, that they use none of their senses, that they neither taste, nor smell what they eat, that they do not eat of themselves, nor do they seek for any thing; in a word, they have no wish, recollection, or judgment. The animals that have lost their cerebral lobes, have lost all their sensations, all their instincts, all their intellectual faculties, and consequently, the unavoidable inference is, that all the instincts, sensations, and intellectual faculties, reside in this part of the brain, exclusively.

These difficulties being removed, another question naturally arises, viz., whether these faculties, instincts, and sensations, since they occupy the same organ, all occupy the same seat, or whether they are distributed, each to its peculiar location.

In the second memoir, the author shows, that however gradual the ablation might be, and in whatever manner it might be conducted, the effect upon all the faculties, sensations, and instincts, was simultaneous, and that they were impaired and lost together. Consequently, the whole, combined, constitute one faculty, and reside in one organ.

In this chapter the author also shows that the cerebrum, the cerebellum, and the tubercula quadrigemina may lose a considerable portion of their substance, without losing the exercise of their functions. A result still more important is, that they may perfectly reacquire these faculties after they have been totally lost.

Another question of considerable importance, and agitated since the days of Hippocrates, is settled here; viz: what parts of the nervous system affect the same, and what the opposite side of the body. The result of the author's experiments is, that the cerebrum, the cerebellum, and the tubercles, have an effect on the opposite side, and that the medulla spinalis and oblongata, affect the same side: from the combination of these different effects, and by the lesion of these different parts, we may explain all the possible cases of paralysis, whether they arise on the same side, with the injury, or on the opposite or whether they are or are not accompanied with convulsions.

Lastly, the author has proved, by an immense number of experiments, that these laws prevail in every class of vertebrated animals.

These are the points most important for their novelty and interest. They are supported in the work before us, by an infinite number of experiments, performed upon almost every variety of animal, within the reach of the author. Before detailing these experiments, we shall translate a part of the report of the committee of the *Academic pour les sciences physiques.*

This committee, consisting of Portal, Count Berthollet, Pinel, Dumeril, and Cuvier, after commenting upon some of the preceding parts of the work, in which we are less interested, proceeds thus:

"That we may better understand the facts which the author has ascertained, we will proceed, in a few words, to give a general description of these parts, and of their mutual relations to each other.

"M. Flourens has pierced the hemispheres of the brain, without producing contraction of the muscles, or any appearance of pain in the animal. He has elevated them by successive incisions, he has performed the same operations upon the cerebellum, and on some occasions he has removed both the cerebrum and the cerebellum, and the animal has in the mean time rested undisturbed. The optic thalami have been attacked in like manner without more effect. The iris did not contract, nor was it paralyzed. But when he pierced the tubercula quadrigemina, there was a commencement of trembling and convulsions, and these phenomena increased in proportion as he advanced deeper into the spinal marrow. The puncture of these, as well as of the optic nerve, produced striking and prolonged contractions of the iris.

"These experiments correspond with those of Lorry. 'Neither the irritations of the brain,' says this physician, 'nor those of the corpus callosum, produce convulsions. The only part of the brain which has appeared capable, of uniformly and universally, exciting convulsions, is the medulla oblongata. It is this which produces them to the exclusion of all other parts.'

"They contradict the experiments of Haller and Zinn, so far as the cerebellum is concerned. But from what M. Flourens has seen, and has exhibited to us, it appears that these experimenters must have touched the medulla oblongata, without being aware of it.

"M. Flourens has concluded that the medulla oblongata and the tubercles possess (to use his own language) irritability; in other words, that they are conductors of irritation, like the spinal marrow and nerves, but that neither the brain nor cerebellum possesses this property. The author has also concluded that the tubercles are a continuation, and the superior termination of the medulla spinalis, and oblongata; and this conclusion is supported by the similarity of their functions, and their anatomical connexions.

"Wounds of the brain and cerebellum produce neither pain nor convulsions, and it is hence concluded, to use the ordinary language, that they are insensible. M. Flourens says that they are the sensitive part of the nervous system, meaning simply, that the impression made upon the sensitive organs must be transmitted to them before the animal experiences a sensation.

"M. Flourens appears to us to have fully proved this proposition, by a reference to the success of sight and hearing. When one cerebral lobe is removed, the animal loses the sight of the opposite eye, although the iris of this eye preserves its mobility, and when both lobes are removed, the animal becomes both blind and deaf.

"But we do not think the same has been sufficiently proved in relation to the other senses. He has made, and could make, no experiment touching the sense of taste and smell; and as to the touch, his experiments do not appear to us conclusive. It is very true, that the animal thus mutilated, puts on the air of sleep, appears to have no volition, makes no voluntary movements; but if struck or pricked, it acts like an animal awake. In whatever position it is placed, it preserves its equilibrium. Laid on its back, it turns over, and walks if it is pushed. If a frog, it swims when touched; and if a bird, it flies on being thrown into the air, struggles when confined, and swallows water that is poured into its beak.

"It would certainly be difficult to believe, that all these actions were produced without sensation. It is true that they are not the effect of reason. The animal moves without design, he is void of memory, and repeatedly opposes himself to the same obstacle. This proves that, in the words of M. Flourens, the animal is in the condition of one asleep, or acts like one asleep; but we would be very far from saying, that a man who moved in his sleep, and knew to choose the most convenient position, could be void of sensation. That the perception is indistinct, and is immediately forgotten, is not proof that they do not exist. Thus, instead of saying with the author, that the cerebral lobes are the only organ of sensation, we limit ourselves by the facts observed, and content ourselves with saying that these lobes are the only receptacle by which the sensation of seeing and hearing can be complete, and become perceptible to the animal. If we wished to add any thing to this, we would say that in them the sensations receive their distinct form, and leave a permanent impression; that they serve as the place for the memory, and thus furnish to the animal the materials out of which its judgments are formed. This conclusion becomes the more probable, because independent of the confirmation it receives from the structure of the lobes, and their connections with the rest of the system, comparative anatomy furnishes additional proof, in the constant proportion which the size of these lobes bears to the degree of intelligence possessed by different species of animals.

"After these effects of the ablation of the cerebral lobes, M. Flourens has examined those of the tubercula quadrigemina.

The removal of these, causes blindness and involuntary motion of the opposite eye. If both are removed, the blindness is complete, and the motion of the eye more violent. Nevertheless, the animal preserves all its faculties, and the iris contracts. The profound section of the tubercles, or the section of the optic nerve, paralyzes the iris; from which M. Flourens concludes that the ablation of the tubercle produces an effect correspondent to the section of the nerve, and that this tubercle is but a conductor of vision, and that the cerebral lobe is the termination of the sensation, and the place where it is perfected and converted into a perception.

"It is to be remarked, that when the section of the tubercles is carried too deeply, it interferes with the spinal marrow, and gives rise to violent and prolonged convulsions.

"But the most curious and novel of the experiments of M. Flourens, are those which relate to the functions of the cerebellum.

"At the commencement of the ablation of this organ, a little weakness and want of harmony in the movements is manifested.

"As the section proceeds it manifests a more general agitation, the animal continuing to see and hear, makes hasty and irregular movements. The faculty of flying, walking, and of standing upright, is lost by degrees; and when the organ is entirely removed, the power of making regular motions is entirely gone.

"Placed on its back, it cannot rise; it sees the blow that menaces it, cries out, seeks to avoid it, and makes a thousand efforts for the purpose, without success. In a word, it preserves the faculty of perception, and volition, without the power of making the muscles obey its will. It scarcely succeeds in supporting itself upright, by supporting itself with the assistance of its wings and tail. By depriving an animal of its brain, it is placed in the condition of sleep; by depriving of the cerebellum, in the condition of intoxication.

"It is surprising (says M. Flourens) to see a pigeon, in proportion as it loses its cerebellum, lose gradually the power of flying, then that of walking, and lastly of supporting itself upright, losing this last by degrees. It commences with being unable to support itself perpendicularly upon its legs, then its feet become insufficient for its support, and at last every fixed position becomes impossible; it makes incredible efforts to arrive at some such position, without success. And nevertheless, when, exhausted with fatigue, it appears to wish for repose, its sensations are so perverted, that the least motion causes it to recommence its contortions, without ever assuming the least convulsive movement, provided the medulla oblongata or the tubercles are untouched.

"We do not remember that any physiologist has pointed out any thing resembling these singular phenomena. Experiments upon the cerebellum of quadrupeds, and especially of adults, are attended with great difficulty, on account of the strong bony structure to be raised, and the blood vessels to be met with.—The most of experimenters, moreover, operate to support some previously conceived opinion, and see with little difficulty what they wish; but certainly no person will now doubt that the cerebellum serves, in some degree, to balance and regulate the locomotion of the animal. This discovery, if these experiments, repeated with the requisite precautions, shall be generally established, will confer very great honor upon this young operator.

"In conclusion, we think, that independent of unnecessary changes of language, and of known facts which the author was compelled to introduce, to give his work a systematic character, this memoir offers, respecting many ancient facts, more precise details than we were before in possession of, and that it contains others, as novel as they are important to science.

"The integrity of the cerebral lobes is necessary to sight and hearing, and when they are removed, the will no longer produces spontaneous movements. Nevertheless, when the animal is excited, it moves as if it sought, at the moment, to avoid pain or uneasiness; but these motions conduce to no object, very probably, because the memory, which has disappeared with the lobes that form its seat, affords no foundation or elements, for the judgment. These movements are not persevered in for the same reason, because the impression which causes them, leaves neither recollection nor permanent volition. The integrity of the cerebellum is necessary to locomotion; while the brain subsists, the animal will see, hear, and have volitions very apparent and energetic; but if the cerebellum is removed, it cannot preserve the equilibrium necessary for locomotion. Irritability subsists a long time in parts without the brain or cerebellum being necessary to it. Every irritation of a nerve brings in action the muscles among which it is distributed. Every irritation of the spinal marrow puts in action the members situated below the point irritated. It is in the spinal marrow, throughout its whole length to the point where it terminates in the tubercula quadrigemina, that the faculty resides, of receiving and propagating irritations, on the one hand, and pain on the other. At this point at least, sensations must arrive in order to their being perceived, and from this point the commands of volition must set out.—Also the continuity of the nervous communication from this point to its distribution, is necessary to voluntary motion, or sensation, whether external or internal.

"These conclusions are not identical with those of the author, nor are they expressed in his language. But they are those which appear to us the most certainly to result from the facts which he has so well established. They will be sufficient to enable you to judge of the importance of the facts, and to induce you to testify your satisfaction, and to request him to continue to communicate the result of a labor so full of interest.

(Signed)

Portal, &c.

Baron Cuvier, Reporter."

After this detail of the opinions of the author, we might here perhaps with propriety close this article. But as the work has not been translated, and as the experiments are full of interest and novelty, we cannot resist the temptation of placing one or two of them, at length, before our readers.

The following experiments were made after the above report, in reply to some objections which it contained, to some of the author's conclusions.

"I concluded from my former experiments respecting the functions of the cerebral lobes, that these lobes are the only receptacle of the sensations.

"There were wanting, to render my position complete and irrefutable, first, direct experiments on the senses of taste and smell, and secondly, more conclusive experiments upon the sense of touch.

"Every person will at once see the extreme importance of preserving alive, for some time at least, the animals submitted to the experiment of extirpating the brain, in order to obtain the full benefit of the experiment. Of the loss of some senses, we may be immediately assured, but the loss of others can be ascertained only by closer observation. Thus it is certain that an animal deprived of the cerebral lobes can neither see nor hear, but how shall we be convinced that it does not smell or taste?

"If an animal, surviving an operation for several months, uses none of his senses, it is clear that he has lost them. If he does not smell, nor perceive the vicinity of food, it is because he has lost the sense of smell, and if the taste does not excite him to swallow food that is placed upon the tongue, or within the beak, it is because he has lost his taste.

"*Experiment*—I removed the two cerebral lobes of a fine and vigorous hen.

"This hen lived afterwards six entire months, in perfect health, and would have lived longer if, at the moment of my return to Paris, I had not been obliged to abandon it.

"During the whole of this time, I did not lose sight of her for a single day, but passed many hours of each day in making observations upon it; studying its habits, and noting its actions; and the following are the observations with which this long study has furnished me.

"Scarcely had I removed the lobes, before she suddenly lost the sight of both eyes. She heard nothing, and gave no signs of volition; but she stood upright, on her legs; she walked when pushed or irritated, she flew when thrown into the air, and swallowed water when it was poured into her beak; but she moved only when irritated. When placed on her feet, she rested on her feet; when laid on her belly, in the position in which these animals repose when asleep, she rested in that position. Constantly was she plunged into a kind of stupor, which neither noise, nor light, but only positive irritations, such as pinching, blows, &c., could interrupt.

"Six hours after this operation, the hen took the attitude of a profound sleep, turning her neck backwards, and concealing her head under the feathers on the upper side of the wing. After leaving her in this position a quarter of an hour, I shook her roughly, and she started from her sleep, but scarcely was she awake, before she fell again into the same situation.

"Eleven hours after the operation, I fed my hen—opening her mouth and filling it with food, she swallowed it very well.

"The next day, the hen awoke a little from her sleep, and with all the actions which all these animals usually make on such an occasion. She shook her head, agitated her plumes, arranging her feathers and cleansing them with her beak, sometimes changing her foot, for she often slept on one foot, as birds not unfrequently do.

"In corresponding cases, one would say of a man asleep, who, without waking entirely, and half-asleep still, changes his place, reposes himself in another situation less fatiguing, stretches his limbs, &c., that he still sleeps.

"The third day, the hen was not as calm as usual; she walked about, but without motive or design; and, if she met with an obstacle, had not sense to avoid it, or turn back. She bore every mark of a violent fever, and I gave her large quantities of water. There was however no sign of convulsions, no irregularity in her motions, and two days afterwards, there was no sign of disease or fever. She became calm, and slept as usual.

"I omit many articles in my journal, and come at once to the second month. The hen enjoyed perfect health, and as I nourished her with great care, she became very fat. She sleeps always very much, and when she is not perfectly asleep, is drowsy.

"For several days past, the bony fragments exposed to the air have been exfoliating and falling off, and the cicatrix is healing rapidly.

"Five months after the operation.—I have never seen a fowl more fat. The wound of the skull is entirely healed, and a fine skin, white and smooth, covers the surface, and beneath this skin, an osseous covering which, although thin, is nevertheless solid.

"I have left this hen for three entire days, and have then placed grain at her nostrils; I have filled her beak with food, have plunged her beak under the water, and have placed her under heaps of corn. She has not smelled, swallowed, nor drunk; she has remained immoveable under the heap of corn, and would certainly have died of hunger, if I had not returned to my former practice of making her eat, myself.

"Twenty times, instead of grain, I have put pebbles into her mouth, and she has swallowed them as if they were grain.

"Finally, this hen has frequently encountered an obstacle with her feet: she strikes against it, and the shock arrests and staggers her, but to strike against a body is not to feel. She never feels, nor gropes, nor hesitates in her walk. ☞ Thus, then, the hen, without the cerebral lobes, has really lost, with the sight and hearing, the smell, taste, and touch. Nevertheless, no one of these senses, or to speak more accurately, no one of the organs of these senses, has been directly injured. The eye is perfectly clear, and the iris moveable. Neither the organ of hearing, nor smell, nor taste, nor touch, has been touched.—Curious fact! There are no sensations, although the organs of sense remain entire. Is it not clear, therefore, that in these organs sensation resides?

"She has lost all her instincts; for she eats nothing, to whatever fasting you expose her; she rejects nothing to whatever excess you may feed her, she never defends herself against other fowls, she knows neither to flee nor to contend; there are no sexual propensities, for the caresses of the male are indifferent or unperceived.

"She has lost all intelligence, for she neither wishes, nor remembers, nor judges."

"I shall add to this case, that of a hen, rendered blind by the extirpation of the tubercula quadrigemina.

"When completely deprived of sight, this hen, for three or four days after the operation, went and came, heard, recollected, sought nourishment, selected it every evening, about the same hour, clambered up on the same table to rest, received the caresses of the male with pleasure, and in walking, advanced with so much precaution, that she seemed to perceive the objects before she touched them.

"She pecked while walking about, and in pecking recognized a good grain, and swallowed it—the bad she rejected.

"She recollected very well the places where her food was usually placed, and the hour of feeding; and she did not fail to repair to the spot when hungry. If the food was displaced, she never rested until she had found the new situation in which it was placed.

"In a word, this curious animal conducted herself, under all circumstances, with an intelligence, the more refined, the more persevering, and the more manifest, in consequence of having lost her sight; being compelled, in consequence of this loss, to supply this defect with all the resources which the other senses, guided by her intellectual faculties, could supply.

"Nothing proves better than these two cases, the contrast, perfect in all respects, between the hen deprived of her tubercles, and the one deprived of the brain; that in losing the first, nothing is lost but the sight, and that in losing the second, or the contrary, all the sensations, and all the intellectual faculties are destroyed.

"To submit this contrast to a new proof, I extirpated the two lobes from two pigeons, and the tubercula quadrigemina from two others. These four pigeons I placed in the same apartment, which I took care to have well supplied with provisions. The two first pigeons died of hunger, the other two knew well where to find their food, to select, and to eat.

"I removed from a hen the right cerebral lobe, and immediately she lost sight of the left eye; I then removed the left tubercle, and she lost the sight of the right eye.

"This hen, thus rendered blind by the extirpation of the cerebral lobe on one side, and of the tubercle on the other, survived three months. She heard well, gave evident signs of intelligence, of volition, governed by reason; she walked but seldom, and when urged to walk, stepped with extreme circumspection, her head stretched, her neck fixed, and her whole body attentive to the slightest impression of external bodies. She also knew where to find her nourishment, and to select it; consequently, she could feed herself.

"I could have objected to the preceding experiments, that they are not conclusive, as to the sense of smell, since the olfactory nerves in which this sense resides, always lose their branches by the complete extirpation of the lobes of the brain.

"I therefore removed the two lobes of the brain from a hen, guarding, with great care, the inferior layers of these lobes, which the branches of the olfactory nerves adhere. This hen thus mutilated, lived for six months, and during this time, I paid great attention to the experiment, without being able to ascertain in her conduct, the least evidence of the existence of this sense.

"This hen neither pecked, nor ate, nor saw, nor heard, nor gave any indication of a will, nevertheless, a sudden impression always roused her; and this impression, if sufficiently strong, produced movements regular and connected.

"But these movements, however regular they might be, were always without object, and without result, either, because the impression, from the want of the cerebral lobes, was not converted into distinct sensation, or perception, as I suppose; or M. Cuvier appears inclined to think, the impression, although perceived, did not leave a recollection, or a lasting impulse, very probably, because the memory which disappears with the lobes, that are its seat, did not furnish the foundation nor the elements for the judgment to act upon.

"It is known that birds almost always seize their food by the extremity of their beak, before eating; but not only do birds deprived of their cerebral lobes, not do this, but they do not eat at all; they do not even peck.

"It is also known, that carnivorous animals, have a habit of running from side to side, smelling every thing; but those that have lost their cerebral lobes, do not smell at all.

"When an animal uses none of its senses, it is to be inferred that it does enjoy them. An animal does not see, when it dashes against every thing it meets; it does not hear, when no noise moves it; it does not smell, when no odour attracts or repels it; it does not taste, when no taste pleases or disgusts it; it does not feel, nor enjoy the sense of touch, when it distinguishes no obstacle, but dashes indiscriminately against all. An animal which feels a body, must, of consequence, form a judgment, and conversely, an animal which does not judge, does not feel.

"Animals deprived of their cerebral lobes, therefore, have neither sensation, nor judgment, nor recollection, nor will; for they cannot will without judgment, nor judge without recollection, nor recollect without having had a sensation. The cerebral lobes, therefore, are the exclusive seat of all the sensations, and all the intellectual faculties.

"The following experiments are intended to settle the question, whether all these faculties occupy concurrently the same seat in these organs, or whether they have each a distinct location.

"I removed from a pigeon, by successive and careful sections, all the anterior portion of the right cerebral lobe, and all the superior and middle portion of the left. The right was gradually weakened, as I advanced, and was not entirely destroyed, until the removal of the layers, near the centre of the two lobes; but the moment that the sight was destroyed, the hearing was lost also; and with the sight and hearing, all the intellectual and sensitive faculties.

"I removed from another pigeon, by successive and careful sections, all the exterior and posterior of the two cerebral lobes, even to some lines of the substance of the corpus callosum. In proportion as the ablation advanced, the sight was gradually and sensibly weakened; the hearing was impaired with the sight, the other faculties with these, and when one was entirely destroyed, they all were.

"The experiment in which the extirpation was commenced, from above, resembled the preceding in its consequences. Consequently, a considerable portion may be cut away from any part of the brain, without destroying its functions. A limited portion of these lobes, therefore, suffice for the exercise of their functions.

"In proportion as the ablation proceeds, all the functions are impaired, and when certain limits are passed, they are all at once destroyed. The cerebral lobes, therefore, concur throughout their whole substance, in the full and entire exercise of all their functions.

"Since when, one of the faculties is lost, all are lost; and when one of the sensations is lost, all are lost, there cannot be different seats for the different faculties or sensations. The faculty of perceiving, of judging, and of wishing one thing, resides in the same place with that of perceiving, judging, and wishing another thing; and, consequently, this faculty, essentially one, resides in one single organ.

"The different organs of the senses have, nevertheless, a distinct origin in the brain. It has been seen already, that the principle of the action of the retina, and of the play of the iris, proceeds from the tubercles. In like manner, the senses of smell, taste, and sight, have their peculiar origin in the particular parts of the brain, which gives rise to their nerves. We may therefore, by destroying each of these separate origins, destroy all the senses, and again, on the contrary, we may destroy, if not all the senses, at least all perception of them, by the destruction of the central organ, while these sensations are consummated.

"We have already shown, that it is possible to remove a certain portion of the lobes of the brain, without entirely destroying their functions. We shall now show that they may recover their functions, after they have been completely destroyed.

"Experiment 1. I removed from a pigeon the central part of the two lobes of the brain, by gradual cuts, stopping as soon as I saw that the senses and intellectual faculties were destroyed.

"The first day, the two mutilated lobes swelled enormously, but the swelling subsided on the second day, and on the third, had nearly disappeared.

"The pigeon commenced from that time to recover by degrees, its sight, hearing, judgment, volition, and other faculties. In about six days it had recovered them all, and what should not be omitted, as it recovered one, it recovered the whole.

2. "On another pigeon, I carried the mutilation a little farther. The animal lost, as in the preceding case, all the sensitive and intellectual faculties, but never recovered them perfectly.

3. "On a third pigeon, I carried the mutilation still further, and all the faculties were destroyed, without returning.

"Thus it appears, that if the destruction of the lobes does not exceed certain limits, the lobes recover after a time, the exercise of their functions; if carried beyond these limits, they recover imperfectly, and if carried still further, they never recover. Further, when one sensation returns, all return, and when one faculty is renewed, all are renewed.

"I removed, by successive sections, all the superior half of the cerebellum of a young cock.

"The animal lost immediately its firmness, and the regularity of its movements, and walked in a staggering and ludicrous manner.

"Four days afterwards, the equilibrium was less disturbed, and he walked with more firmness and confidence. Five days afterwards, he was perfectly restored.

"I removed from a pigeon, a little more than half of the cerebellum, and cut it away entirely from a hen. After a short time, the pigeon recovered his equilibrium entirely, but the hen did not, although she survived the operation more than four months.

"I removed the superior layers of the tubercula quadrigemina of the right side, of one pigeon, and of the left of another. On the fourth day, each of these pigeons saw a little with the eye that had been lost; the sight improved daily, and after some days, recovered perfectly.

"The tubercles, therefore, and the cerebellum, partake in common with the cerebral lobes, the faculty of recovering their lost faculties, and of recovering them, although they themselves are not entire.

"In conclusion, we may remark, that this perfect destruction of the function of an organ, in consequence of the removal of a part of the organ, and the restoration of the functions in consequence of a part being permitted to remain, proves clearly, that each of these organs forms one simple organ; for the injury of a part, impairs the whole, and the preservation of a part leaves room for the restoration of the whole."

*Wounds of the brain.*—Wounds of the brain readily heal. Passing over the author's experiments, we shall simply give one or two circumstances, which appear to us not destitute of interest.

"Longitudinal wounds of the cerebral lobes, whatever may be their situation and extent, readily heal with an entire restoration of their functions; but transverse sections, in some situations, and of sufficient extent, never heal. All wounds of the cerebellum, on the contrary, heal perfectly. This difference is readily accounted for, by the different manner in which these organs are attached to their crura in animals. The position of the brain is such, that a transverse section may separate one part entirely, and the part thus separated dies. With the cerebellum this can never take place."

M. Flourens asserts, that the restoration of substance, which has been said by some, to take place, in wounds of the brain, never occurs. This error has probably arisen from the enormous tumefaction, which we witness in wounds of the brain—a tumefaction which would lead one to say, that the more we cut off, the more it expands. But as soon as the tumefaction disappears, the parts return to their natural volume, and it may then be clearly seen, that the parts which have been removed are wanting, as long as the animal survives the operation.

The author has made many experiments, for the purpose of ascertaining what parts of the nervous system, influence the same side of the body, and what influence the opposite side. The following is the sum of his observations.

"When one side of the brain or cerebellum is injured, paralysis takes place on the opposite side. If one side of the medulla spinalis or oblongata is wounded, there will be, according to the extent of the injury, paralysis, or convulsions, of the same side; a wound of the tubercula quadrigemina produces convulsions or paralysis of the opposite side.

"When one cerebral lobe and the corresponding side of the medulla oblongata are injured at once, there will be paralysis of the opposite side, and convulsions [or paralysis] of the same side. If one side of the cerebellum, and of the medulla oblongata are wounded, the symptoms will be as before."*

These cases will include all the possible combination of symptoms, which may arise from injury of these parts.

The most important position which the author has established, in this work, is the fact, that the concert of muscles, combining to produce a definite motion, is regulated by the cerebellum. Of this fact there seems to be no doubt, if the experiments can be at all credited. The animal, after the loss of the cerebellum, still retains the power of moving the muscles; but is utterly incapable of combining their movements to any useful purpose.

There are, however, other combined movements, which are not under the control of this organ. The motion of the muscles, supplied by the *respiratory system* of nerves, is of this class. The concert of these muscles is independent of the cerebellum; and the author distinguishes them by the name of *movements of conservation*, and locates the centre of their action, in the spinal marrow. Their motion is produced by a local irritation, acting like that which produces the contraction of the iris, at a dis-

*The symptoms here, are different from what the preceding experiments would lead us to expect, viz: a paralysis of one side, and the loss of the concert of the muscles of the other.

tance from the affected muscle, and differing from the local irritation which produces the involuntary contraction of other muscles, in requiring the communication with the medulla oblongata, to be reserved as the medium through which the sympathetic impression is communicated.

The author has ineffectually endeavoured to arrange, under the same head, (*mouvemens de conservation*,) the muscular efforts, by which the equilibrium of the body is preserved. It was essential to his system, that they should not be considered as voluntary motions, since they take place when the cerebral lobes are removed. The author, we think, has unnecessarily involved himself in a difficulty, from the want of making a proper distinction between volition and the will. The former is, we suspect, purely an animal faculty, having very little connection with the intellect. The faculty of preserving the equilibrium, seems to form a link between the purely voluntary motions, and the respiratory muscles, as these last seem to be placed midway between the voluntary and involuntary muscles.

In taking leave of the author, we cannot avoid remarking the great contrast between the experiments of the author, and those of Mr. Charles Bell, in a similar investigation,—a contrast which prevails very generally, between the investigations of the French and English Physiologists.

In the former, we witness a passion for the knife, amounting to infatuation—as if nature would continue her operations uninterrupted when her recesses were exposed to vulgar observation. The sources of error in this mode of investigation are innumerable, and the difficulties of drawing positive conclusions are so great, that we cannot help being surprised at the air of assurance, with which these gentlemen jump from an experiment to a conclusion. As an instance of this rashness of induction, we would notice the facility with which the author proves that the brain is a simple organ, and that volition, sensation, and intellection, are its functions, having all a common residence. He removes the cranium, and cuts away the brain by slices, encroaching by degrees upon the domicil of wit, humor, fancy, intellect, dwelling in delightful harmony with the social and domestic affections, and doubtless, seriously incommoded with the unwelcome vicinity of the grosser animal appetites. As the enemy encroaches upon the seat, where, snug within the recesses of the cranium, they have heretofore flourished, they pine away and die together; but if, perchance, their habitation should not be rendered perfectly untenantable, they collect again around the family altar, as soon as the first alarm subsides, and apportion the remains of the dilapidated building, with a due regard to the rights of each.

Now, to this summary conclusion, there are some serious objections. First, we have not the convenient mode of ascertaining the existence of intellectual faculties, that Milton's angels possessed, and consequently, if the mutilated chicken was not in a very communicative mood, it would be difficult to say how much they were disturbed by the operation: Secondly, the facts are very inconsistent with what we daily observe in diseases of the brain—mania, partial apoplexy, &c. Lastly, there is very great reason to doubt whether this gradual loss of the mental faculties may not be owing to the sympathetic influence of such severe wounds, and consequently whether the experiment may not be entirely inapplicable.

We confess that we are among those who think that this wanton infliction of animal suffering is to be deprecated; nor can we believe that experiments of wanton and unnecessary cruelty, can be conducted without injury to the social virtues.

We question very much whether the labors of Bichat and Legallois, in this field of observation, have advanced very materially the cause of physiological science, and still less do we believe that, if their contributions to the science had been much greater than they are, that the ultimate objects of science would have been at all advanced by them.

What have we gained by the millions of animals which the disciples of this school have submitted to tortures, from which every unsophisticated person turns with horror. Prompted by the love of novelty, we may tolerate a few judicious experiments of this kind, in an original mind that can turn them to good account. But, the information we gain by them is extremely limited, indefinite, and uncertain, and but poorly compensates for the labor and time bestowed upon them; and when the few principles to which they lead are established, it argues neither good sense nor good taste unnecessarily to repeat them.

